# Infection of RANKL-Primed RAW-D Macrophages with *Porphyromonas gingivalis* Promotes Osteoclastogenesis in a TNF-α-Independent Manner

**DOI:** 10.1371/journal.pone.0038500

**Published:** 2012-06-18

**Authors:** Akiko Kukita, Yuka Ichigi, Ippei Takigawa, Toshiyuki Watanabe, Toshio Kukita, Hiroshi Miyamoto

**Affiliations:** 1 Department of Microbiology, Faculty of Medicine, Saga University, Saga, Japan; 2 Molecular Cell Biology & Oral Anatomy, Faculty of Dental Science, Kyushu University, Fukuoka, Japan; Charité-University Medicine Berlin, Germany

## Abstract

Infection of macrophages with bacteria induces the production of pro-inflammatory cytokines including TNF-α. TNF-α directly stimulates osteoclast differentiation from bone marrow macrophages *in vitro* as well as indirectly via osteoblasts. Recently, it was reported that bacterial components such as LPS inhibited RANKL-induced osteoclastogenesis in early stages, but promoted osteoclast differentiation in late stages. However, the contribution to osteoclast differentiation of TNF-α produced by infected macrophages remains unclear. We show here that *Porphyromonas gingivalis*, one of the major pathogens in periodontitis, directly promotes osteoclastogenesis from RANKL-primed RAW-D (subclone of RAW264) mouse macrophages, and we show that TNF-α is not involved in the stimulatory effect on osteoclastogenesis. *P. gingivalis* infection of RANKL-primed RAW-D macrophages markedly stimulated osteoclastogenesis in a RANKL-independent manner. In the presence of the TLR4 inhibitor, polymyxin B, infection of RANKL-primed RAW-D cells with *P.* gingivalis also induced osteoclastogenesis, indicating that TLR4 is not involved. Infection of RAW-D cells with *P. gingivalis* stimulated the production of TNF-α, whereas the production of TNF-α by similarly infected RANKL-primed RAW-D cells was markedly down-regulated. In addition, infection of RANKL-primed macrophages with *P. gingivalis* induced osteoclastogenesis in the presence of neutralizing antibody against TNF-α. Inhibitors of NFATc1 and p38MAPK, but not of NF-κB signaling, significantly suppressed *P. gingivalis*-induced osteoclastogenesis from RANKL-primed macrophages. Moreover, re-treatment of RANKL-primed macrophages with RANKL stimulated osteoclastogenesis in the presence or absence of *P. gingivalis* infection, whereas re-treatment of RANKL-primed macrophages with TNF-α did not enhance osteoclastogenesis in the presence of live *P. gingivalis*. Thus, *P. gingivalis* infection of RANKL-primed macrophages promoted osteoclastogenesis in a TNF-α independent manner, and RANKL but not TNF-α was effective in inducing osteoclastogenesis from RANKL-primed RAW-D cells in the presence of *P. gingivalis*.

## Introduction

Osteoclasts are large multinucleated cells that are derived from the common progenitor cells of the monocyte/macrophage lineage. Osteoclasts play a central role in bone resorption. Bone remodeling involves cooperation of osteoclasts and bone forming osteoblasts to maintain normal bone volume and calcium homeostasis. However, in inflammatory diseases such as periodontal disease and rheumatoid arthritis, the balance between osteoclasts and osteoblasts is disrupted, and osteoclasts accumulate causing local pathological bone loss.

Differentiation of osteoclasts is regulated by macrophage-colony-stimulating factor (M-CSF) and receptor activator of nuclear factor κB ligand (RANKL) [1]. RANKL, which belongs to the tumor necrosis factor (TNF) family, binds to the TNF family receptor, receptor activator of nuclear factor κB (RANK) expressed on osteoclast precursor cells, and triggers osteoclast differentiation. The signaling mechanisms underlying RANKL-induced osteoclastogenesis have been extensively studied [2]. Binding of RANKL to RANK leads to the activation of NF-κB and mitogen activated protein kinases (MAPKs), including p38 and c-Jun N-terminal kinase (JNK). Deletion of both of the NF-κB subunits p50 and p52 results in defective osteoclast differentiation and causes osteopetrosis in mice, indicating that the NF-κB pathway controls osteoclastogenesis [3]. c-Fos is also activated by RANK and plays an essential role in osteoclast differentiation [4]. NF-κB and c-Fos induce the expression of nuclear factor of activated T cells cytoplasmic 1 (NFATc1), which is auto-amplified during osteoclastogenesis [5]. NFATc1 regulates the expression of osteoclast-specific genes including calcitonin receptor, cathepsin K, and tartrate-resistant acid phosphatase (TRAP) [6]. RANK signaling also induces the expression of genes that inhibit osteoclast differentiation; e.g., IFN-β induced by RANKL inhibits osteoclastogenesis by suppressing c-Fos expression [7].

Periodontitis is a common inflammatory disease, and bone destruction in periodontitis is usually associated with bacterial infections [8], [9]. LPS, a major constituent of Gram-negative bacteria and an important TLR4 ligand, has been shown to be a potent stimulator of bone loss *in vivo* [10]. However, TLR ligands have been shown to have stimulatory or inhibitory functions on osteoclastogenesis *in vitro*. Simultaneous addition of RANKL and LPS or staphylococcal lipoteichoic acid inhibits differentiation of osteoclasts from bone marrow macrophage (BMM) [11], [12]. LPS, in contrast, stimulates the late stage of osteoclastogenesis and enhances the survival and activation of osteoclasts [11], [13], [14]. Very recently, Zhang *et al* reported that *P. gingivalis,* which is implicated in periodontitis, differentially affects osteoclast differentiation from bone marrow macrophages depending on the stage of osteoclast differentiation [15]. In contrast, TLR ligands promote osteoclastogenesis via other cells such as osteoblasts. *E. coli* LPS and diacyl lipoprotein stimulate the expression of RANKL and IL-6 in osteoblasts through TLRs, and promote osteoclastogenesis in co-cultures of osteoblasts and hematopoietic cells [16], [17], [18]. LPS also stimulates the production of PGE2 in osteoblasts, which leads to bone resorption [19].

Down-stream signaling pathways of TLRs, other than TLR3, utilize myeloid differentiation factor 88 (Myd88). Myd88 recruits IL-1R-associated kinases leading to the activation of NF-κB and MAPK. Activated NF-κB then induces the transcription of inflammatory genes such as TNF-α and IL-6 [20], [21]. *P. gingivalis* is a Gram-negative bacterial species, but its LPS has a unique chemical structure, and interacts with both TLR2 and TLR4. *P. gingivalis* LPS weakly activates TLR4 signaling, and its biological activities are primarily mediated via signaling through TLR2 [22]. On the other hand, live *P. gingivalis* induces cytokines and chemokines such as TNF-α, IL-6, and MCP-1, which signal through both TLR2 and TLR4 [22]. TNF-α is known as a major inducer not only of inflammation but also of bone loss. TNF-α directly acts on BMM exposed to RANKL or transforming growth factor (TGF)-β, and induces osteoclast differentiation in a RANKL independent manner *in vitro* [23], [24]. TNF-α is a multifunctional cytokine, and also participates in restraining inflammation and tissue healing [25], [26]. Thus, the role of TNF-α in osteoclastogenesis in bacterial infection is not obvious.

We have previously demonstrated that a macrophage cell line, RAW-D, a subclone of RAW264 has a high capacity to differentiate into osteoclasts [27]. In the present study, we investigated the effect of infection of RAW-D with *P. gingivalis* on osteoclastogenesis. Our results demonstrate that infection with *P. gingivalis* markedly stimulated osteoclast differentiation from RANKL-primed RAW-D cells. We found that osteoclastogenesis induced by *P. gingivalis* infection of RANKL-primed RAW-D cells and BMM was TNF-α independent, and we found that RANKL but not TNF-α was effective in inducing osteoclastogenesis from RANKL-primed RAW-D cells in the presence of *P. gingivalis*.

## Results

### Infection of RANKL-primed RAW-D Cells with *P. gingivalis* Induces Osteoclastogenesis

**Figure 1:**
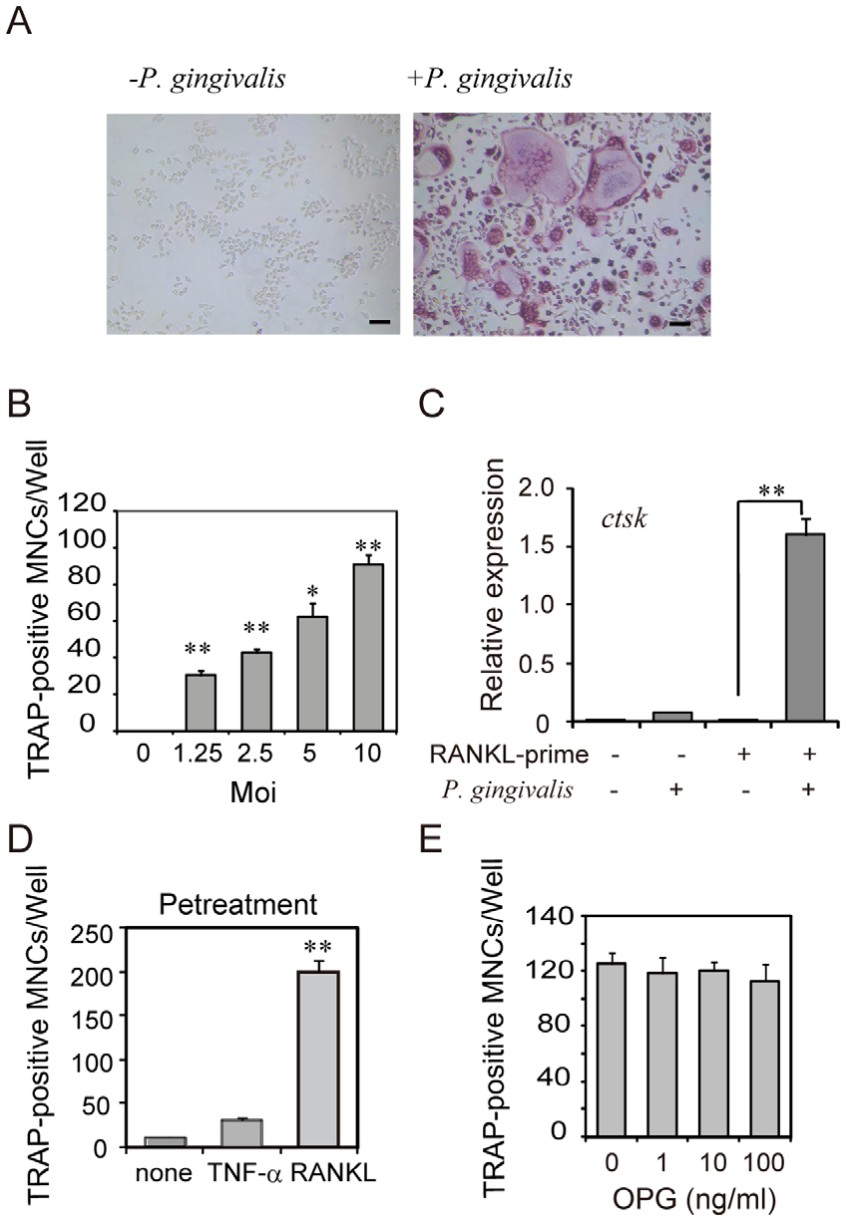
Infection of RANKL-primed RAW-D macrophages with *P. gingivalis* induces osteoclastogenesis. (A) (B) *P. gingivalis* infection of RANKL-primed RAW-D cells induces the formation of TRAP-positive MNCs. (C) *P. gingivalis* infection of RANKL-primed RAW-D cells induces mRNA expression of the osteoclast-specific gene, cathepsin K. Total RNA was isolated, and cathepsin K expression was assessed by real-time PCR. Expression levels were normalized to GAPDH. Effect of pretreatment (D), or OPG (E) on osteoclastogenesis induced by infection with *P. gingivalis*. RAW-D cells were primed with RANKL (50 ng/ml) or TNF-α (10 ng/ml) for 22 h, then infected with *P. gingivalis*, and cultured for 24–48 h. After 24 h, RNA was extracted and analyzed for gene expression. After 48 h, the culture was stained for TRAP, and TRAP-positive MNCs were counted. Data are expressed as mean ± S.D. of four independent cultures. Statistical significance was determined with Student’s *t* test. **P<0.01, *P<0.05 compared to uninfected control (B, C) or untreated control (D).

We first examined whether *P. gingivalis* infection induced osteoclastogenesis in a mouse macrophage cell line, RAW-D. Although RAW-D has a high potential to differentiate into osteoclasts, *P. gingivalis* infection alone did not induce osteoclastogenesis in RAW-D cells (data not shown). Because recent studies have shown that LPS stimulates osteoclast differentiation from RANKL-pretreated osteoclast precursors [14], we stimulated RAW-D cells with RANKL for 22 h, then removed the RANKL, and infected the cells with *P. gingivalis.* Cells were cultured for two more days, and the effect of *P. gingivalis* infection on osteoclast differentiation was analyzed. After the initial 22 h of culture in the presence of RANKL, i.e., after RANKL-priming, a few mononuclear cells positive for the osteoclast-specific enzyme TRAP were present, but no TRAP-positive multinucleated cells (MNCs) were observed, and no TRAP-positive MNCs appeared during further culture for 48 h in the absence of RANKL and *P. gingivalis* (Fig. 1A, left). In contrast, infection of RANKL-primed RAW-D cells with *P. gingivalis* induced osteoclastogenesis in an infectious dose-dependent manner (Figs. 1A right, and 1B). We analyzed mRNA expression levels of several osteoclast-specific genes in unprimed or RANKL-primed RAW-D cells that were infected with *P. gingivalis* or were uninfected. *P. gingivalis* infection of RANKL-primed RAW-D cells significantly increased the expression of osteoclast-specific genes such as cathepsin K (*ctsk*) (Fig. 1C) and calcitonin receptors (*calcr*) (data not shown) in comparison with uninfected RANKL-primed RAW-D cells. Pretreatment with TNF-α instead of RANKL did not induce osteoclast differentiation (Fig. 1D). Osteoprotegrin (OPG) did not inhibit osteoclastogenesis induced by infection with *P. gingivalis* (Fig. 1E). Thus, RANKL-pretreatment was necessary, but concurrent presence of RANKL was not required for osteoclastogenesis in RANKL-primed macrophages induced by infection with *P. gingivalis*. These data indicate that infection of RANKL-primed macrophages with *P. gingivalis* induced osteoclast differentiation from osteoclast precursor cells.

### TLR4 is not Involved in Osteoclastogenesis Induced by the Infection of RANKL-primed RAW-D Cells with *P. gingivalis*

**Figure 2:**
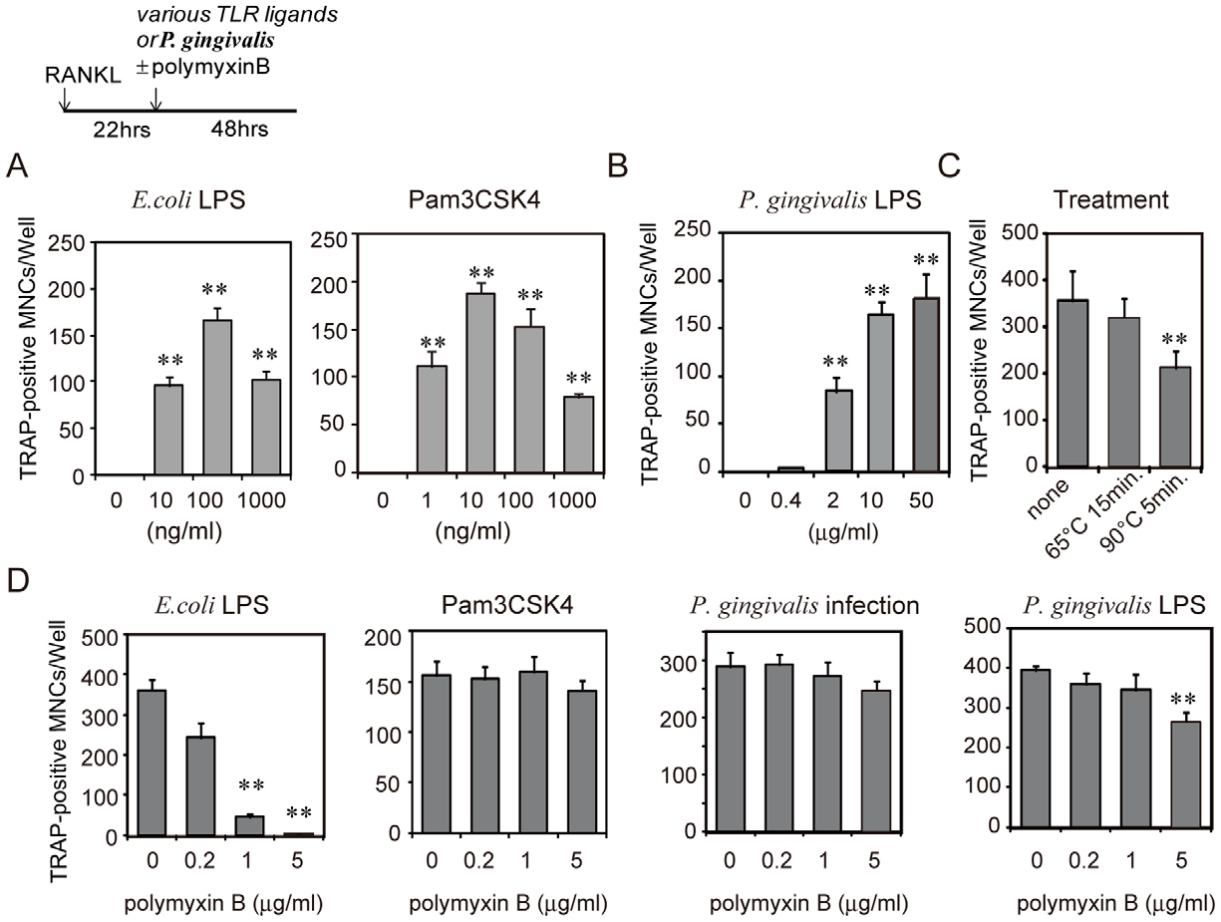
TLR4 is not involved in osteoclastogenesis in RANKL-primed RAW-D cells induced by infection with *P. gingivalis*. Effect of *E.coli* LPS or Pam3CSK4 (A) or *P. gingivalis* LPS (B) on osteoclastogenesis in RANKL-primed RAW-D cells. (C) Effect of heat treatment of *P. gingivalis* on osteoclastogenesis in RANKL-primed RAW-D cells. (D) Effect of polymyxin B on osteoclast formation in RANKL-primed RAW-D cells induced by *E. coli* LPS, Pam3CSK4, live *P. gingivalis*, or *P. gingivalis* LPS. RAW-D cells were primed with RANKL (50 ng/ml) for 22 h and then treated with *E. coli* LPS (100 ng/ml), Pam3CSK4 (100 ng/ml), *P. gingivalis* LPS (10 µg/ml) or live *P. gingivalis* (m.o.i.  = 10) in the presence of various concentrations of polymyxin B. After 48 h, the culture was stained for TRAP, and the number of TRAP-positive MNCs was counted. Data are expressed as mean ± S.D. of four independent cultures. Statistical significance was determined with Student’s *t* test. **P<0.01 compared to untreated controls (A, B, and C) or controls without polymyxin B (D).

*P. gingivalis* is known to stimulate the production of TNF-α and IL-6 through TLR2 and TLR4 signals [22]. Therefore, we analyzed TLRs involved in the stimulation of osteoclastogenesis induced by *P. gingivalis* infection. Treatment with *E. coli* LPS, a TLR4 ligand, and the synthetic lipoprotein Pam3CSK4, a TLR2 ligand, stimulated osteoclastogenesis in RANKL-primed RAW-D cells (Fig. 2A). Similarly, *P. gingivalis* LPS induced osteoclastogenesis in RANKL-primed RAW-D cells (Fig. 2B). We found that the concentration of *P. gingivalis* LPS required to stimulate osteoclastogenesis was higher than the concentration of *E. coli* LPS required for similar stimulation. *P. gingivalis* treated at 65°C for 15 min stimulated osteoclastogenesis at levels similar to live *P. gingivalis*, but treatment of *P. gingivalis* at 90°C for 5 min reduced the induction of osteoclastogenesis from RANKL-primed RAW-D cells (Fig. 2C), suggesting that some protein components of live *P. gingivalis* may be involved. Polymyxin B (1 µg/ml), which is a specific inhibitor of TLR4, inhibited osteoclastogenesis in RANKL-primed RAW-D cells induced by *E. coli* LPS, but not in cells induced by Pam3CSK4. However, the same concentration of polymyxin B (1 µg/ml) did not inhibit the induction of osteoclastogenesis in RANKL-primed RAW-D cells induced by live *P. gingivalis* or *P. gingivalis* LPS (Fig. 2D). Although a higher concentration of polymyxin B (5 µg/ml) partially inhibited the induction of osteoclastogenesis in RANKL-primed RAW-D cells by *P. gingivalis* LPS, these results show that the major effect of *P. gingivalis* on osteoclastogenesis in RANKL-primed RAW-D cells does not involve TLR 4 signaling.

### TNF-α Induces Osteoclastogenesis in RANKL-primed Macrophages, but Infection with *P. gingivalis* Induces Osteoclastogenesis in RANKL-primed Macrophages in the Absence of TNF-α

**Figure 3:**
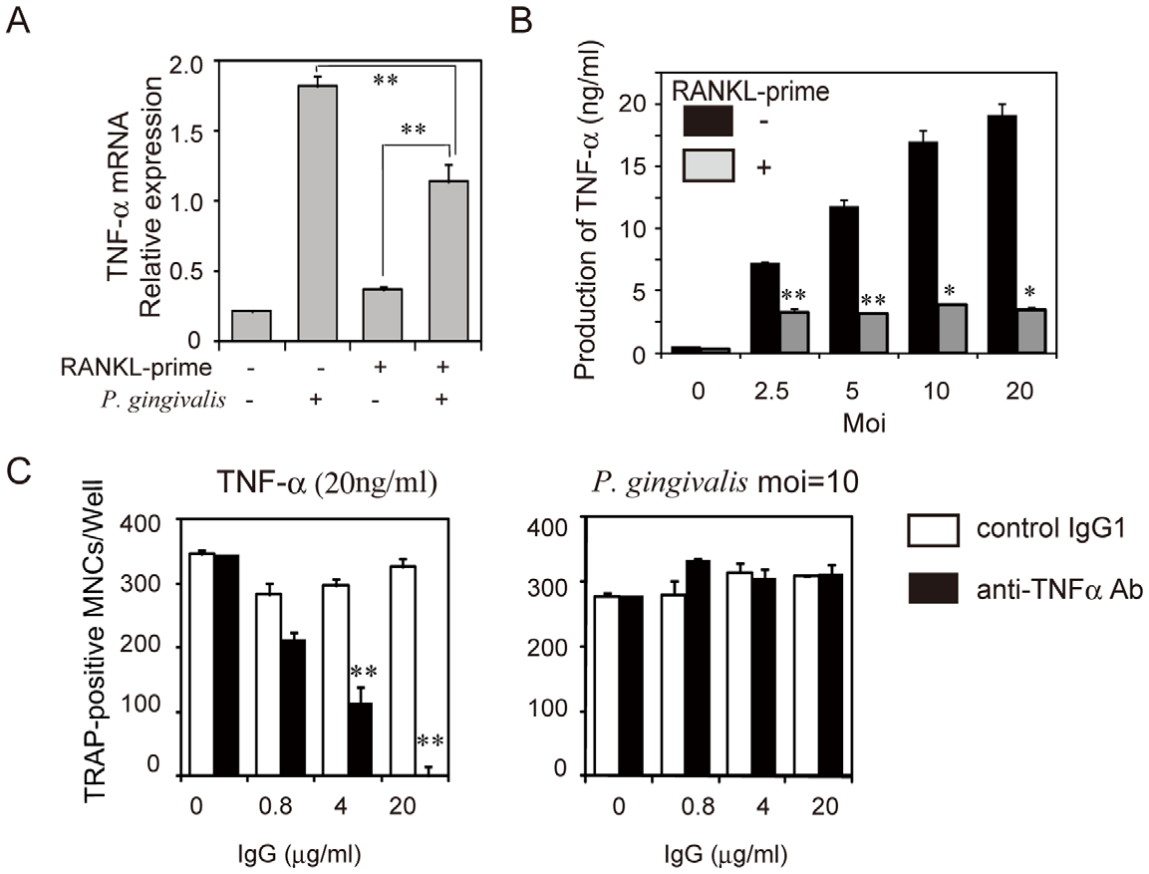
*P. gingivalis* induces osteoclastogenesis in RANKL-primed RAW-D cells in the absence of TNF-α. Analysis of TNF-α mRNA expression (A) or production of TNF-α protein (B) by *P. gingivalis* infected RANKL-primed RAW-D cells and unprimed cells. (C) Effect of neutralizing antibody against mouse TNF-α on osteoclast formation in RANKL-primed RAW-D cells induced by TNF-α or live *P. gingivalis*. RAW-D cells were primed with RANKL (50 ng/ml) for 22 h and then retreated with TNF-α or live *P. gingivalis* in the presence or absence of neutralizing antibody against mouse TNF-α or control IgG. After 24 h, RNA was extracted, and TNF-α mRNA expression was assessed by real-time PCR. After 48 h, cell supernatants were collected and analyzed for TNF-α by ELISA. After 48 h, the culture was stained for TRAP, and the number of TRAP-positive MNCs was counted. Data are expressed as mean ± S.D. of four independent cultures. Statistical significance was determined with Student’s *t* test. **P<0.01, *P<0.05 compared with unprimed infected RAW-D or RANKL-primed uninfected control (A), unprimed control (B), or control IgG1 (C).

TNF-α has been shown to induce osteoclast differentiation in mouse BMM *in vitro* [24]. To analyze the contribution of TNF-α to osteoclastogenesis in RANKL-primed RAW-D cells infected with *P. gingivalis,* we analyzed TNF-α mRNA expression. Infection of unprimed or RANKL-primed RAW-D cells with *P. gingivalis* stimulated the expression of TNF-α. However, level of TNF-α induced in RANKL-primed RAW-D cells was only 60–65% of the level induced in unprimed RAW-D cells (Fig. 3A). In addition, the production of TNF-α protein from RANKL-primed RAW-D cells induced by *P. gingivalis* was markedly decreased (Fig. 3B). Because addition of TNF-α stimulated osteoclastogenesis in RANKL-primed RAW-D cells (Fig. 3C), we analyzed the capacity of *P. gingivalis* infection to induce osteoclastogenesis in RANKL-primed RAW-D in the presence of neutralizing antibody against mouse TNF-α. TNF-α neutralizing antibody completely blocked osteoclast differentiation in uninfected RANKL-primed RAW-D cells in the presence of mouse TNF-α (20 ng/ml) (Fig. 3C, left). In contrast, TNF-α neutralizing antibody did not block osteoclast differentiation in *P. gingivalis*-infected RANKL-primed RAW-D cells (Fig. 3C, right). These data indicate that TNF-α is not required for osteoclastogenesis in RANKL-primed RAW-D induced by infection with *P. gingivalis*.

**Figure 4:**
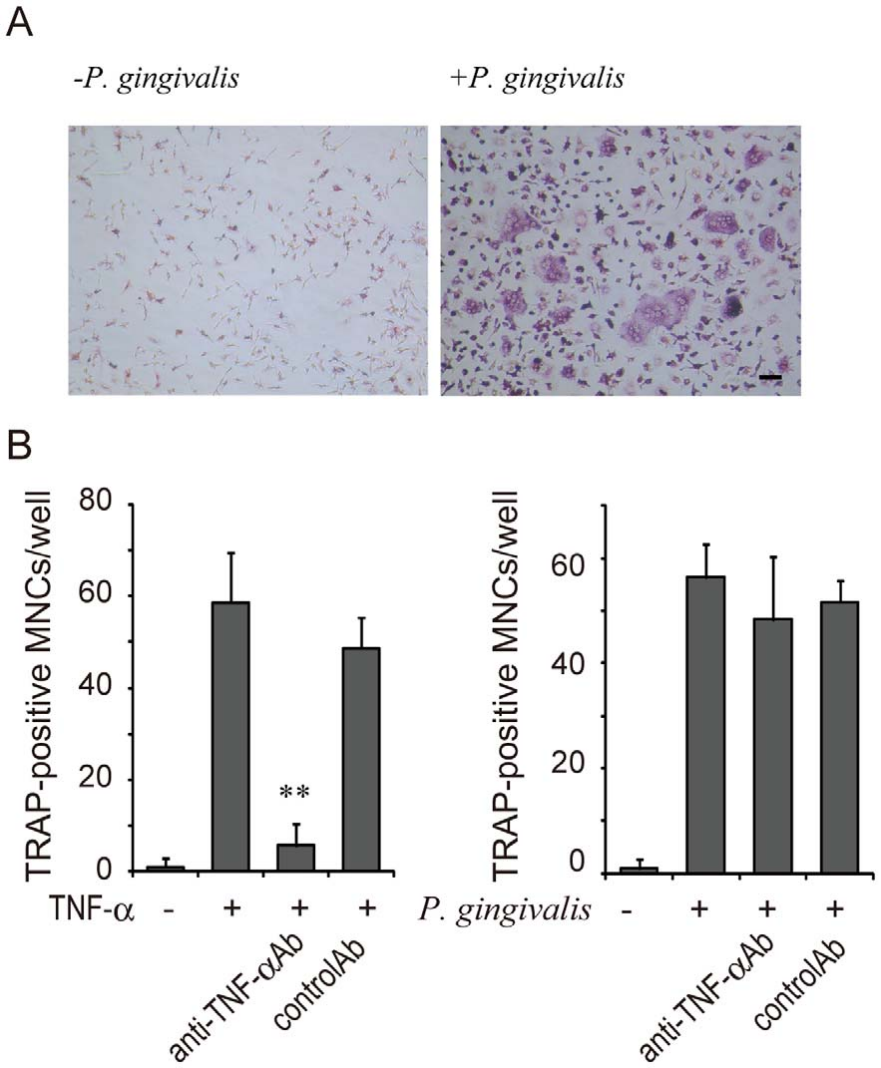
Infection of RANKL-primed BMM with *P. gingivalis* induces osteoclastogenesis in the absence of TNF-α. (A) Infection of RANKL-primed BMM with *P. gingivalis* induces osteoclastogenesis. Representative photographs are shown. (B) Effect of neutralizing antibody against mouse TNF-α on osteoclastogenesis in RANKL-primed BMM induced by TNF-α or live *P. gingivalis*. BMM were stimulated with RANKL (50 ng/ml) for 22 h and then re-stimulated by TNF-α or live *P. gingivalis* (m.o.i.  = 10) in the presence or absence of neutralizing antibody against mouse TNF-α or control IgG. At the end of culture, the culture was stained for TRAP, and TRAP-positive MNCs were counted. Data are expressed as mean ± S.D. of four independent cultures. Statistical significance was determined with Student’s *t* test. **P<0.01, compared with control IgG1.

We next analyzed mouse BMMs to determine whether TNF-α is involved in osteoclast differentiation in RANKL-primed macrophages in primary culture. We found that, as was the case in RANKL-primed RAW-D cells, *P. gingivalis* infection of RANKL-primed BMM stimulated osteoclastogenesis (Fig. 4A). Neutralizing antibody against anti-mouse TNF-α blocked osteoclast differentiation induced by TNF-α but did not inhibit osteoclast differentiation induced by infection of RANKL-primed BMM with *P. gingivalis* (Fig. 4B).

### Expression of NFATc1 but not c-fos is up-regulated During Osteoclastogenesis in RANKL-primed RAW-D Cells Induced by Infection with *P. gingivalis*

**Figure 5:**
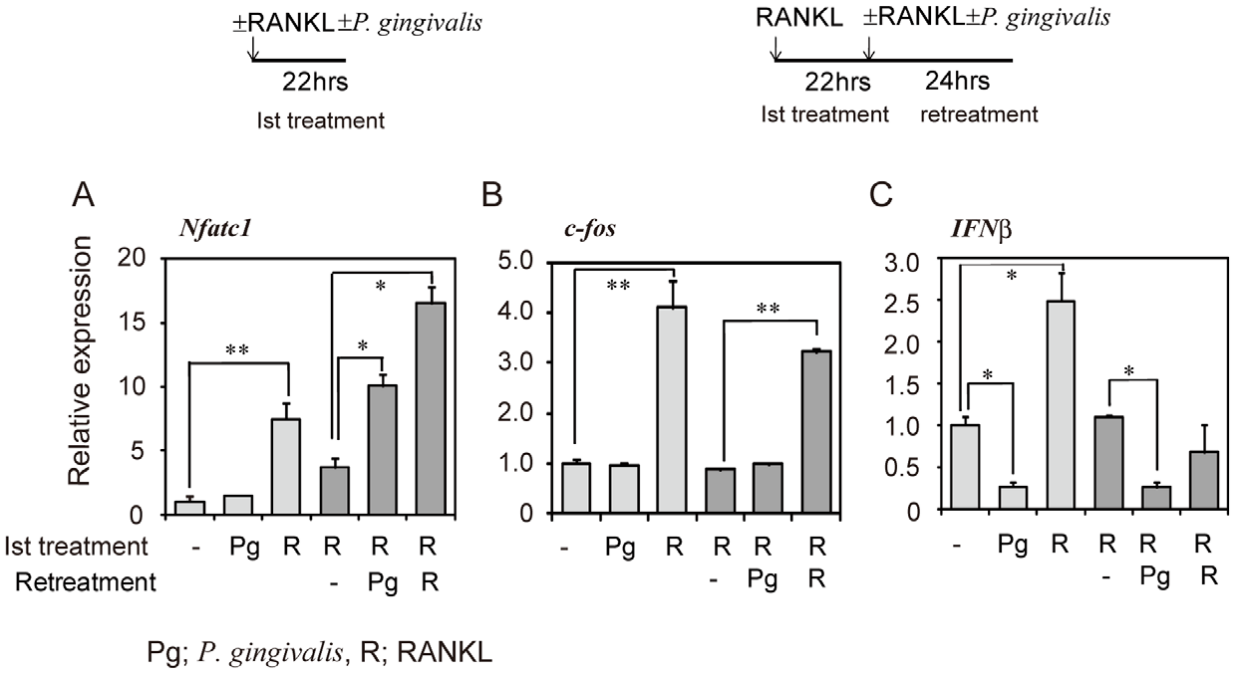
Expression of osteoclast signaling proteins in osteoclastogenesis in RANKL-primed RAW-D cells induced by infection. RAW-D cells were stimulated with or without RANKL (50 ng/ml) or *P. gingivalis* for 22 h. RANKL-primed RAW-D cells were then retreated with or without RANKL (50 ng/ml) or *P. gingivalis* for 24 h. Total RNA was prepared, cDNA was synthesized, and real-time PCR analysis was performed using NFATc1 (A), c-fos (B), or IFNβ (C) Taqman probes. Statistical significance was determined with Student’s *t* test. **P<0.01, *P<0.05 compared with unprimed control or RANKL-primed control.

To assess the contribution of osteoclast signaling molecules in osteoclastogenesis in RANKL-primed RAW-D cells induced by infection with *P. gingivalis*, we analyzed mRNA expression levels of NFATc1, c-Fos, and IFNβ in unprimed RAW-D cells treated or not treated with RANKL or *P. gingivalis*, and in RANKL-primed RAW-D cells retreated or not retreated with RANKL or *P. gingivalis*. Initial RANKL treatment of RAW-D cells stimulated the expression of both NFATc1 and c-fos. However, expression of both NFATc1 and c-fos declined if the cells were further cultured in the absence of RANKL or *P. gingivalis* for 24 h (Fig. 5A and B, compare bars 3 and 4). Retreatment of RANKL-primed macrophages with RANKL increased expression of both NFATc1 and c-fos compared to unretreated cells. In contrast, infection (i.e., retreatment) of RANKL-primed cells with live *P. gingivalis* increased expression of NFATc1 but not of c-fos (Fig. 5A and B). INFβ is known to inhibit osteoclastogenesis by down-regulating c-Fos [7]. RANKL stimulated the expression of IFNβ, whereas *P. gingivalis* infection of RAW-D cells reduced the expression of IFNβ. Retreatment of RANKL-treated RAW-D cells with *P. gingivalis* also decreased the expression of IFNβ mRNA (Fig. 5C). Together, these data suggest that expression of NFATc1 is important in osteoclastogenesis in RANKL-primed RAW-D cells induced by infection with *P. gingivalis*, and the down-regulation of IFNβ expression induced by *P. gingivalis* may facilitate induction of osteoclast differentiation.

### NFATc1 and p38 MAPK Signaling but not JNK or NF-κB Signaling are Required for Osteoclastogenesis in RANKL-primed RAW-D Cells Induced by Infection with *P. gingivalis*

**Figure 6:**
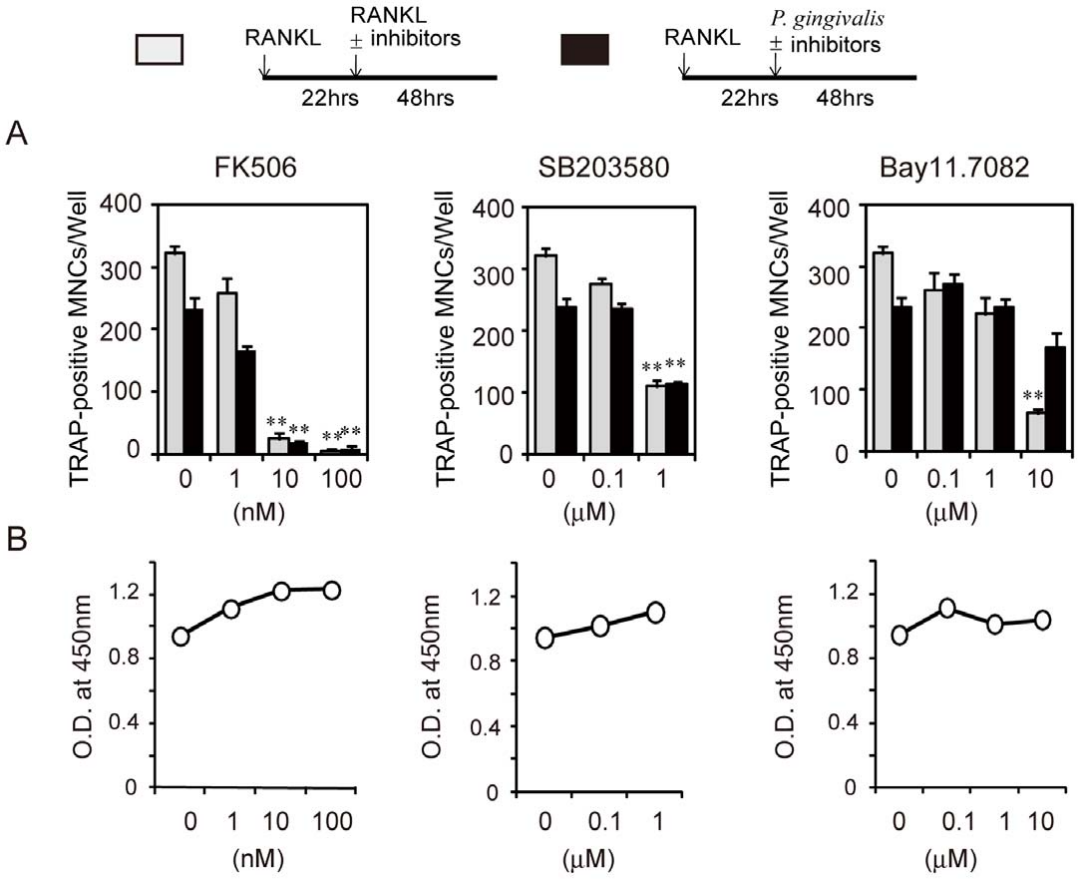
Effect of signaling inhibitors on osteoclastogenesis in RANKL-primed RAW-D cells induced by infection. (A) Effect of inhibitors NFAT (FK506), p38 MAPK (SB203580), and NF-κB (Bay11.7082) on osteoclastogenesis in RANKL-primed RAW-D cells induced by RANKL or by infection with *P. gingivalis*. (B) Effect of inhibitors on cell proliferation of RANKL-primed RAW-D cells in the presence of *P. gingivalis* for 2 days. RAW-D cells were stimulated with RANKL (50 ng/ml) for 22 h and then retreated with RANKL or *P. gingivalis* in the presence or absence of various concentrations of inhibitors. The culture was stained for TRAP activity after 48 h and TRAP-positive MNCs were counted. Proliferation was analyzed using CCK-8 cell proliferation kit. Data are expressed as mean ± S.D. of four independent cultures. Statistical significance was determined with Student’s *t* test. **P<0.01 compared to cultures without inhibitors.

To elucidate the mechanism of osteoclast induction, we analyzed the effect of inhibitors of NFATc1, MAPK, and NF-κB pathways on osteoclastogenesis in RANKL-primed RAW-D cells induced by infection with *P. gingivalis*. All inhibitors suppressed osteoclastogenesis in RANKL-primed RAW-D cells induced by retreatment with RANKL (Fig. 6A). Inhibitors of NFATc1 (FK506) and p38 MAPK (SB203580), similarly inhibited osteoclastogenesis in RANKL-primed RAW-D cells induced by infection with *P. gingivalis* (Fig. 6A). In contrast, inhibitor of NF-κB (Bay11.7082) did not significantly inhibit osteoclastogenesis in RANKL-primed RAW-D cells induced by infection with *P. gingivalis*. The concentrations of these inhibitors used in the differentiation assays did not inhibit the proliferation of RAW-D cells (Fig. 6B). These results indicate that osteoclastogenesis in RANKL-primed RAW-D cells induced by infection with *P. gingivalis* is dependent on the expression of NFATc1 and p38 MAPK but is not dependent on expression of NF-κB, whereas osteoclastogenesis induced in these cells by RANKL is dependent on NF-κB as well as on NFATc1 and p38 MAPK.

### RANKL but not TNF-α Enhances Osteoclastogenesis in RANKL-primed RAW-D Cells in the Presence of *P. gingivalis*

**Figure 7:**
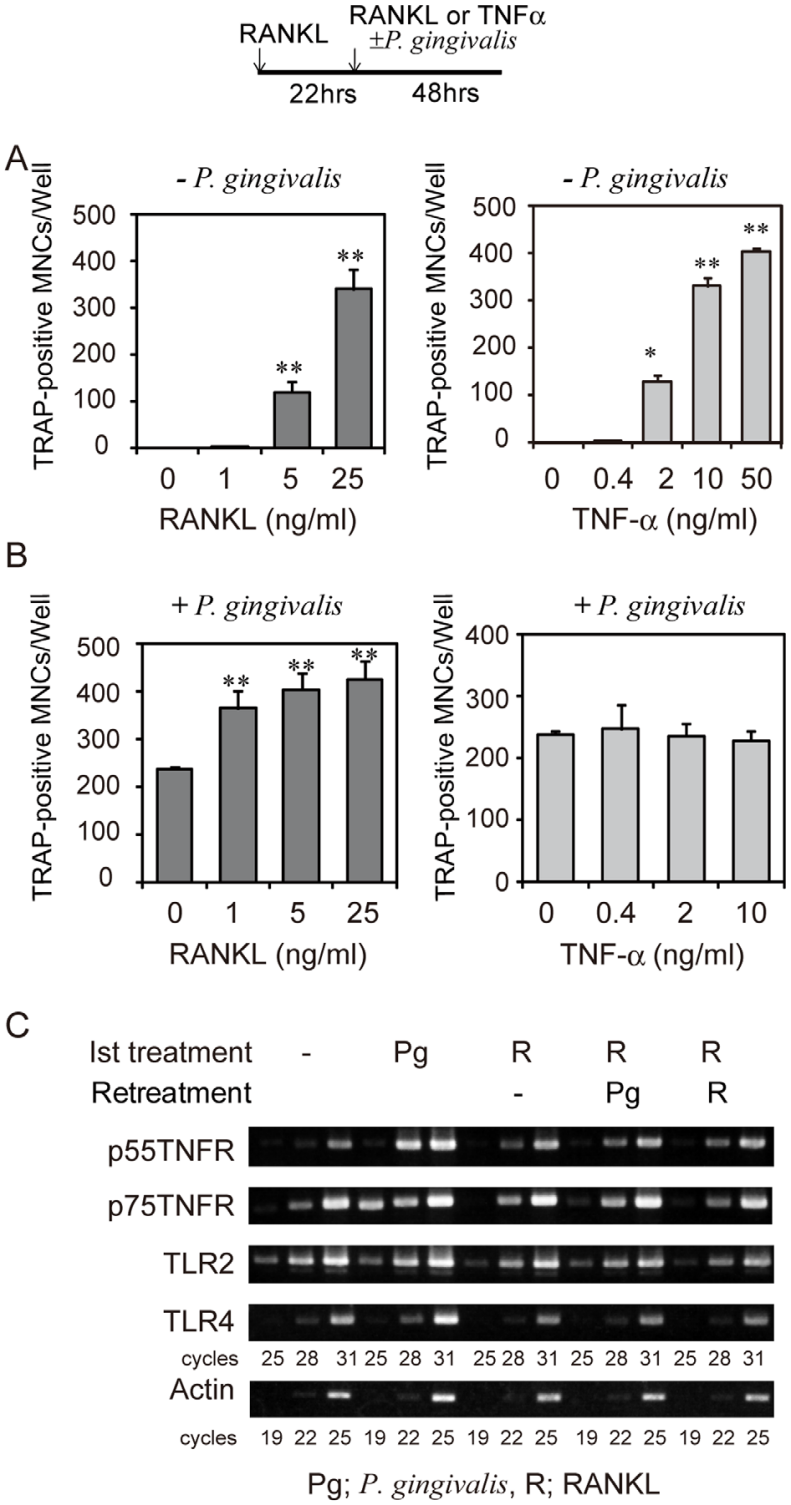
RANKL but not TNF-α promotes osteoclastogenesis in RANKL-primed RAW-D cells in the presence of *P. gingivalis*. RAW-D cells were stimulated with RANKL (50 ng/ml) for 22 h and then re-stimulated with RANKL or TNF-α in the absence (A) or presence (B) of live *P. gingivalis.* The culture was stained for TRAP activity after 48 h of retreatment, and TRAP-positive MNCs were counted. Data are expressed as mean ± S.D. of four independent cultures. Statistical significance was determined with Student’s *t* test. **P<0.01, compared to cultures without RANKL or TNF-α. (C) Expression of mRNAs for p55 and p75 TNF receptors, TLR2, and TLR4 in RAW-D cells treated with or without *P. gingivalis* for 22 h, or RANKL-primed RAW-D cells retreated with or without RANKL or *P. gingivalis* for 24 h. Total RNA was isolated, and mRNA expression was assessed by semi-quantitative RT-PCR using specific primers as described in *Materials and Methods*.

To determine the role of RANKL and TNF-α in osteoclastogenesis induced by infection with *P. gingivalis*, we further analyzed the effect of RANKL and TNF-α on osteoclastogenesis in RANKL-primed RAW-D cells in the presence or absence of *P. gingivalis*. RANKL retreatment stimulated osteoclastogenesis in a dose-dependent manner in RANKL-primed RAW-D cells (Fig. 7A, left). RANKL retreatment coupled with live *P. gingivalis* infection further enhanced osteoclastogenesis (Fig. 7B, left). Similarly, TNF-α retreatment of RANKL-primed RAW-D cells strongly stimulated osteoclast formation in RANKL-primed RAW-D cells (Fig. 7A, right). However, TNF-α retreatment coupled with live *P. gingivalis* infection did not increase osteoclast formation (Fig. 7B, right). These data indicate that TNF-α stimulates osteoclastogenesis in osteoclast precursor cells in the absence, but not in the presence, of *P. gingivalis*. On the other hand, *R*ANKL enhances osteoclastogenesis in osteoclast precursor cells both in the presence or absence of *P. gingivalis*.

We analyzed mRNA expression of p55 TNF receptor, p75 TNF receptor, TLR2, and TLR4 in RANKL-primed or untreated RAW-D cells and in RANKL-primed RAW-D cells retreated with *P. gingivalis* or RANKL by semi-quantitative RT-PCR. Expression of p55 and p75 TNF receptors was increased by *P. gingivalis* and was not down-regulated in RANKL-primed RAW-D cells. Expression of both TLR2 and TLR4 was also detected in RANKL-primed RAW-D cells (Fig. 7C). These data suggest that the inability of TNF-α to enhance osteoclastogenesis in RANKL-primed RAW-D in the presence of *P. gingivalis* may not be a consequence of the loss of expression of TNF-α receptors.

## Discussion

Osteoclasts are responsible for inflammatory bone loss caused by diseases such as periodontitis and rheumatoid arthritis. Periodontitis is a common infectious inflammatory disease associated with resorption of alveolar bone. It has previously been reported that bacterial components such as *E. coli* LPS induce bone loss *in vivo* and affect osteoclastogenesis *in vitro* [10], [16], [17], [18]. Recent studies have shown that *E. coli* LPS inhibits osteoclastogenesis from bone marrow macrophages in early stages of the process but stimulates osteoclastogenesis in later stages. [14]. Hotokezaka *et al.* have also shown that peptidoglycan in addition to *E. coli* LPS induce osteoclastogenesis and especially cell fusion in RANKL-treated RAW264.7 cells in the presence of a low concentration of U0126, a MAPK-ERK kinase (MEK)/ERK inhibitor [28]. While our experiments were ongoing, Zhang *et al* reported that *P. gingivalis* differentially modulates osteoclast differentiation from bone marrow macrophages depending on the stage of osteoclastogenesis [15]. However, it is possible that, if the adherent cells are not positively depleted, primary bone marrow macrophage preparations will be contaminated with small numbers of other cell lineages such as T cells and stromal cells that may affect osteoclast differentiation [23]. Direct effects of pathogens such as *P. gingivalis* on osteoclastogenesis are still not clear. In addition, although cytokines are considered to be involved in the pathology of periodontitis [29], [30], the contribution of TNF-α produced by macrophages to osteoclastogenesis is obscure. In the present study, using the RAW-D macrophage cell line, we demonstrated that infection of RANKL-primed macrophages with *P. gingivalis* markedly promoted osteoclastogenesis in the absence of TNF-α.

Zhang *et al.* showed that *P. gingivalis* increases osteoclastogenesis two-fold compared to RANKL alone as assessed by analysis of the formation of TRAP-positive MNCs. We found a more marked effect of *P. gingivalis* infection on osteoclastogenesis from RANKL-primed RAW-D cells. If RANKL-primed RAW-D cells were cultured without stimulation, they did not form TRAP-positive cells, whereas, if RANKL-primed RAW-D cells were infected with *P. gingivalis* they formed numerous TRAP-positive MNCs. We also showed evidence that *P. gingivalis* infection induces expression of several osteoclast-specific genes such as cathepsin K and calcitonin receptors. As we used a macrophage cell line, RAW-D, it is apparent that the effect of *P. gingivalis* infection is the result of direct action on RAW-D cells. We also showed that *P. gingivalis* LPS similarly affects osteoclast induction in RANKL-primed RAW-D macrophages.

Cytokine production from macrophages infected with *P. gingivalis* is down-regulated in both TLR2 and TLR4 knockout mice, although the reduction is greater in TLR2 knockout mice [15], [22]. On the other hand, it has been shown that TLR2 is crucial for inflammatory bone loss in an experimental model of periodontitis induced by infection with *P. gingivalis* [31]. Zhang *et al.* showed the importance of TLR2 and Myd88 in the inhibition of RANKL-induced osteoclastogenesis by *P. gingivalis* infection in unprimed bone marrow macrophages. In this study, using polymyxin B, we showed that TLR4 was not necessary for the induction of osteoclastogenesis by *P. gingivalis* infection. These results suggest that TLR2 is involved in osteoclastogenesis in RANKL-primed RAW-D cells induced by *P. gingivalis* infection. We also found that a high amount of *P. gingivalis* LPS was necessary to induce osteoclastogenesis, indicating that the signal through *P. gingivalis* LPS is weak or other components of *P. gingivalis* may be involved. Heat treatment of *P. gingivalis* reduced the stimulatory activity suggesting that some protein components may be involved. Several virulence factors of *P. gingivalis* such as LPS, lipoproteins, Fim A fimbriae, hemagglutinins, and cysteine proteinases (gingipains) are considered to have pathological roles in periodontitis [32]. *P. gingivalis* Fim A fimbrial proteins signal through TLR2 and induce the production of inflammatory cytokines in macrophages [22], [33]. However, Fim A proteins have been shown to be activated by heat treatment at 95°C for 10 min [34]. Further investigation is necessary to elucidate the contribution of TLRs in osteoclast formation induced by live *P. gingivalis* in RANKL-primed macrophages.

Using specific inhibitors, we found that different signaling pathways are required for osteoclastogenesis in RANKL-primed RAW-D cells induced by *P. gingivalis* than in cells induced by RANKL. Although inhibitors of both of NFATc1 and NF-κB inhibited osteoclastogenesis induced by RANKL, a specific inhibitor of NFATc1, but not an inhibitor of NF-κB, inhibited osteoclastogenesis induced by infection with *P. gingivalis* in RANKL-primed RAW-D cells. Osteoclastogenesis induced by *P. gingivalis* in RANKL-primed RAW-D cells was dependent on NFATc1 but not NF-κB. Interestingly, Zhang *et al.* showed the existence of a negative feedback loop between NF-ATc1 and NF-κB [15]. In their study, NFATc1 signaling was activated, but *P. gingivalis*-induced NF-κB activation was down-regulated in RANKL-primed macrophages. Although the mechanism is unknown, our results support Zhang *et al.*’s hypothesis. It remains to be determined how NFATc1 is activated in osteoclastogenesis induced by *P. gingivalis* in RANKL-primed macrophages. In osteoclastogenesis, NF-κB and c-Fos are recruited to the NFATc1 promoter and contribute to its transcription in early phases of osteoclastogenesis; however, in later phases NF-κB and c-Fos are not involved as NFATc1 stimulates its own transcription through an auto-amplification loop [35]. Therefore, in RANKL-primed macrophages, it is possible that the expression level of NFATc1is sufficient to auto-amplify its own expression. However, *P. gingivalis* may employ additional mechanisms to maintain the localization and expression of NFATc1. It has been reported that peptidoglycan and LPS activate phospholipase C γ2 (PLCγ2), leading to intracellular calcium mobilization in BMM [36], which may activate NFATc1.

An important observation in the current report is that TNF-α is not required for osteoclastogenesis in RANKL-primed RAW-D cells induced by *P. gingivalis* infection. Several reports have shown that RANKL treatment of macrophages reduces the production of inflammatory cytokines induced by LPS [15], [37]. RANKL pretreatment also protected mice from LPS-induced death from endotoxic shock caused by excess amounts of pro-inflammatory cytokines [38]. In the current study, we have shown that *P. gingivalis* infection stimulates osteoclastogenesis in the presence of neutralizing antibody against TNF-α. In addition, we showed that osteoclastogenesis induced by *P. gingivalis* infection was not significantly inhibited by a specific inhibitor of NF-κB, adding further support to the conclusion that TNF-α is not involved. In contrast, Ukai *et al.* recently reported that culture supernatant from *P. gingivalis*-stimulated macrophages induced osteoclastogenesis from BMM, and TNF-α was involved [39]. Although our data conflict with their results, this discrepancy can be attributed to differences in the culture conditions or presence of live *P. gingivalis* in our experiments. Our finding that TNF-α induces osteoclastogenesis in RANKL-primed RAW-D cells in the absence of stimulation with TLR ligands is similar to the results of Ukai *et al.*

More interestingly, TNF-α did not enhance osteoclastogenesis in RANKL-primed RAW-D induced in the presence of *P. gingivalis.* At present, it is unknown why RANKL-primed macrophages are not responsive to TNF-α in the presence of *P. gingivalis*. This is likely to be the result of differences in the endogenous signaling response of RANKL-primed macrophages to TNF-α in the presence or absence of *P. gingivalis*. It has been reported that some negative regulators of NF-κB activation, such as A20, are induced by LPS [40]. Pretreatment of macrophages with TNF-α suppressed cytokine induction by inhibiting LPS-induced NF-κB signaling; this effect was mediated by A20 and glycogen synthase kinase 3-α (GSK3) [25]. Our results suggest that there may be similar regulatory mechanisms operating between signaling through TNF receptor and TLRs, which may be an important regulatory mechanism for protection from excessive inflammation.

In contrast to TNF-α, retreatment with RANKL promoted osteoclast differentiation from RANKL-primed macrophages in the presence of *P. gingivalis*. It has recently been shown that RANKL antagonists and OPG inhibit bone loss in experimental periodontitis [41], [42], indicating the importance of RANKL and OPG balance in bone loss in periodontitis. In addition, a previous study showed that a higher percentage of T and B cells expressed RANKL in bone resorption lesions of diseased gingival tissues [43]. Consistent with previous results, our data indicate that RANKL plays an important role in bone destruction in the presence of pathogen.

**Figure 8:**
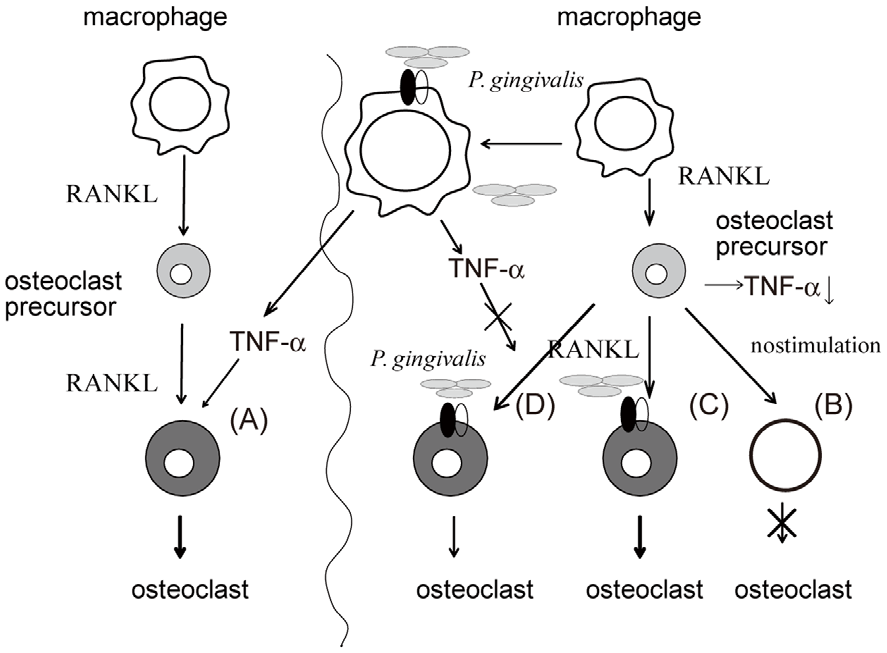
Possible role of TNF-α in osteoclastogenesis in the presence or absence of *P. gingivalis*. Macrophages respond to infection with *P. gingivalis* by producing TNF-α, which stimulates osteoclastogenesis in osteoclast precursor cells in the absence of *P. gingivalis* (A). However, osteoclast precursor cells primed with RANKL do not produce TNF-α and respond differentially to various stimuli. (B) Cells that are not stimulated do not differentiate into osteoclasts. (C) Cells that are continuously re-stimulated with RANKL differentiate into osteoclasts in an NFATc1- and NF-κB-dependent manner in the presence of *P. gingivalis*. (D) Cells that are infected with *P. gingivalis* differentiate into osteoclasts in an NFATc1-dependent and NF-κB-independent manner. TNF-α does not stimulate osteoclastogenesis in osteoclast precursor cells in the presence of *P. gingivalis*, whereas RANKL stimulates osteoclastogenesis in the presence or absence of *P. gingivalis*.

Our current work suggests a possible role of TNF-α and RANKL in osteoclastogenesis induced by infection with *P. gingivalis* (Fig. 8). Infection of osteoclast precursor cells with *P. gingivalis* markedly stimulates osteoclastogenesis in an NFATc1 dependent but NF-κB independent manner. TNF-α has the ability to promote osteoclastogenesis in the absence of direct stimulation with pathogen, but does not promote osteoclastogenesis in osteoclast precursor cells in the presence of *P. gingivalis*. In contrast, RANKL effectively stimulated osteoclastogenesis from osteoclast precursor cells in the presence or absence of *P. gingivalis*. Our results identify differential effects of RANKL and TNF-α on osteoclastogenesis in RANKL-primed macrophages in the presence of pathogen, which will be useful in devising strategies to regulate bone loss in infection-induced inflammatory diseases.

## Materials and Methods

### Materials

FCS was purchased from BioWhittaker (Walkersville, MD). Recombinant human soluble RANKL, M-CSF, and OPG (osteoprotegerin) were from Pepro-Tech (London, United Kingdom). Recombinant mouse TNF-α was purchased from R&D Systems (Minneapolis, MN). LPS from *Escherichia coli* O55:B5 and *Porphyromonas gingivalis* ATCC33277 were purchased from Sigma-Aldrich (St. Louis MO) and InvivoGen (San Diego, CA), respectively. Synthetic bacterial lipoprotein, Pam3CSK4, and polymyxin B were obtained from InvivoGen (San Diego, CA). NF-AT activation inhibitor, FK506, was obtained from LC Laboratories (Woburn MA). P38 MAPK inhibitor, SB203580, was purchased from Calbiochem (San Diego, CA). IκB-α inhibitor, BAY11-8082, was obtained from InvivoGen (San Diego, CA). Neutralizing antibody against anti-mouse TNF-α (MP6-XT3) and isotype control antibody, rat IgG1 (eBRG), were from eBioscience (San Diego, CA). Male C57BL/6 mice (aged 5–10 weeks) were purchased from Kyudo Co. (Saga, Japan).

### Ethics Statement

All experiments were reviewed and approved by the Laboratory Animal Care and Use Committee of Saga University, permit number (20-026-4).

### Bacterial Strain and Culture

*P. gingivalis* ATCC33277 (kindly provided by Dr. K. Nakayama, Nagasaki University, Japan) was cultured and maintained on enriched trypto-soy agar plates containing 5 mg/ml hemine, defibrinated sheep blood, and 1 mg/ml menadione, at 37°C in an anaerobic atmosphere. For the preparation of *P. gingivalis* for cell stimulation, bacteria were harvested, centrifuged, and washed in PBS. The number of bacteria (CFU/ml) was determined by measuring the optical density (OD) at 600 nm.

### Cell Culture and Osteoclastogenesis

The murine macrophage cell line RAW-D (a subclone of RAW264) [27], was cultured in α-MEM containing 10% FCS. For stimulation with TLR ligands and *P. gingivalis*, RAW-D cells were precultured with RANKL (50 ng/ml) for 22 h. The cells were then replated in 96 well plates at a density of 4.5×10^4^ cells/ml. Cells were then infected with live *P. gingivalis* at indicated multiplicities of infection (MOI), or incubated in the presence or absence of RANKL (5 ng/ml) for 1–2 days. For stimulation of primary macrophages, bone marrow cells were cultured in the presence of 10 ng/ml M-CSF for 3 days to generate BMMs, which were then stimulated with 50 ng/ml RANKL for 22 h, then infected with *P. gingivalis* and cultured in the presence of M-CSF (10 ng/ml) for 2 days [5]. At the end of the culture, cells were fixed and stained with a commercial kit for the osteoclast marker, TRAP (Sigma-Aldrich, St. Louis, MO). TRAP-positive cells with 3 or more nuclei were counted as multinucleated cells (MNCs).

### RT-PCR and Real-time PCR

Total RNA was extracted using Isogen (Nippon Gene), according to the manufacturer’s protocol. cDNA was synthesized from 1 µg total RNA using a random primer and avian myeloblastosis virus RT and a PrimeScript RT-PCR kit (Takara Bio, Inc. Shiga, Japan). PCR was performed using Quick HS Taq DyeMix (Toyobo). The following primers were used for semi-quantitative RT-PCR analysis: mouse *tnfrsf1a* (p55 TNF receptor), sense, 5′-GAAGTTGTGCCTACCTCCTC-3′, antisense, 5′-GTGATTCGTAGAGCAGAGGG-3′; mouse *tnfrsf1b* (p75 TNF receptor), sense, 5′-ACGTTCTCTGACACCACATC-3′, antisense, 5′-TGGCATCTCTTTGTAGGCAG-3′; mouse *tlr2* (TLR2), sense, 5′-GCATGGATCAGAAACTCAGC-3′, antisense, 5′-CAACCGATGGACGTGTAAAC-3′; mouse *tlr4* (TLR4), sense, 5′-TCTGATGGTGAAGGTTGGAC-3′, antisense, 5′-CCAAATGTTCAAGACTGCCC-3′. As an internal control for RNA quantity, the same cDNA was amplified using primers specific for mouse actin mRNA: sense, 5′-AGGGTGTGATGGTGGGAAT-3′, antisense, 5′-TGCTATGTTGCTCTAGACTTCGAG-3′. Real-time PCR reactions were performed using a TaqMan gene expression assay kit with a StepOnePlus real-time PCR system (Applied Biosystems Foster City, CA). Reactions were conducted in a 10 µl reaction mixture containing 900 nM primers and 250 nM probes, and were incubated 10 min at 95°C, followed by 40 cycles of a two-step amplification procedure composed of annealing/extension for 1 min at 60°C and denaturation for 15 s at 95°C. mRNA levels were quantified using a standard curve generated with serially diluted cDNA and normalized to *Gapdh* expression. Commercially available probe-primer sets (Applied Biosystems) with proprietary sequences were used.

### Cell Viability Assay

RANKL-primed RAW-D cells were infected with *P. gingivalis*, and cultured in the presence of various concentrations of signaling inhibitors for 2 days. Cell viability was evaluated using Cell Counting kit-8 (CCK-8) (Dojin Laboratories, Japan) reagent. A 1/10 volume of reagent was added to each well, and the cells were incubated at 37°C for an additional 2 h. Absorbance at 450 nm was then measured.

### ELISA Analysis

Culture supernatants were collected, and protein levels of TNF-α were measured by enzyme-linked immunosorbent assay using an ELISA kit (eBioscience, San Diego, CA) according to the manufacturer’s instructions.
